# Determining the status of non-transferred embryos in Ireland: a conspectus of case law and implications for clinical IVF practice

**DOI:** 10.1186/1747-5341-4-8

**Published:** 2009-07-09

**Authors:** Eric Scott Sills, Sarah Ellen Murphy

**Affiliations:** 1 Division of Reproductive Endocrinology and Infertility, Department of Obstetrics & Gynaecology, School of Medicine, Royal College of Surgeons in Ireland, Dublin, Ireland; 2 The Sims Institute/Sims International Fertility Clinic, Dublin 14, Ireland; 3 Department of Law, University College Cork, Cork, Ireland

## Abstract

The development of *in vitro *fertilisation (IVF) as a treatment for human infertilty was among the most controversial medical achievements of the modern era. In Ireland, the fate and status of supranumary (non-transferred) embryos derived from IVF brings challenges both for clinical practice and public health policy because there is no judicial or legislative framework in place to address the medical, scientific, or ethical uncertainties. Complex legal issues exist regarding informed consent and ownership of embryos, particularly the use of non-transferred embryos if a couple separates or divorces. But since case law is only beginning to emerge from outside Ireland and because legislation on IVF and human embryo status is entirely absent here, this matter is poised to raise contractual, constitutional and property law issues at the highest level. Our analysis examines this medico-legal challenge in an Irish context, and summarises key decisions on this issue rendered from other jurisdictions. The contractual issues raised by the *Roche *case regarding informed consent and the implications the initial judgment may have for future disputes over embryos are also discussed. Our research also considers a putative Constitutional 'right to procreate' and the implications EU law may have for an Irish case concerning the fate of frozen embryos. Since current Medical Council guidelines are insufficient to ensure appropriate regulation of the advanced reproductive technologies in Ireland, the report of the Commission on Assisted Human Reproduction is most likely to influence embryo custody disputes. Public policy requires the establishment and implementation of a more comprehensive legislative framework within which assisted reproductive medical services are offered.

## Introduction

Although the status of the 'unborn' has been debated since Aristotle [1,2], the search for clarification has never been more urgent. In delivering judgment on a dispute over the control of non-transferred embryos, Irish courts confront this ancient question in a way that will yield answers aspiring to be definitive, but will probably not receive universal approval. They may look to philosophical debate in order to make an informed decision about the status of the non-transferred embryo compared to the status of the naturally conceived (*in vivo*) embryo and the subsequent protections afforded to it. This judicial landscape is not entirely new: the abortion debate also pivoted on this question, as did the issues of whether the contraceptive pill was ethically acceptable. Irish courts could also be influenced by the debates surrounding the (ultimately successful) Eighth Amendment to the Constitution Bill [3], as this reflected the prevailing electoral opinion on reproductive issues in 1983.

While selecting conception as the beginning of human life has intrinsic appeal due to its simplicity as a discrete, identifiable event, that very characteristic draws criticism [4,5]. This approach means that human embryos conceived either naturally (*in vivo*) or with medical assistance (*in vitro*) would be accorded the same legal rights and protections. However, the non-transferred IVF embryo (in contrast to the *in vivo *embryo) is not necessarily 'on the way to being born'. Indeed, the embryo derived from IVF treatment still requires a significant amount of skill and intervention in order to provide it with the potential to be born. If this is not provided by medical science, and if the embryo is not transferred to the uterus, then the non-transferred embryo will not be born. If this view of the moral status of the embryo were strictly followed, then 'any research or other manipulationthat damages any embryo or interferes with its prospects for transfer to a uterus and subsequent development is ethically unacceptableonce conceived, the being was recognised as man because he had man's potential. The criterion for humanity thus was simple and all-embracing: If you are conceived by human parents, you are human'[6].

The potentiality argument assigns legal rights to the embryo derived from IVF on the basis that it has the *potential *to develop into a human being. This approach admits the uncertainty of pinpointing the exact time when life can be said to begin, and instead advocates that the embryo deserves entitlements because it has the potential for human life. But how should this potentiality position be applied to non-transferred embryos? In one sense, it fails to reconcile the distinction between what *is *and what *could be*. For example, 'the bare fact that something will *become *X (even if it will inevitably become X, which is far from being the case with the fertilised egg and the adult human being) is not a good reason for treating it now as if it *were *in fact X.' For example, anyone now reading these words is potentially dead, but that is hardly justification for treating those individuals as if they were *already *dead [7].

In the UK, the Warnock Report [4,8] concluded that the human embryo obtained from IVF does not have the potential to develop into a human merely because it occupies a petri dish in the laboratory. From this application of the potentiality principle, the IVF embryo has an undeclared (*i.e*., yet to be determined) moral value and may be stored, used for research purposes, or discarded. This position is supported by the recognised high loss rate for human embryos throughout the early stages of pregnancy in nature. Should Irish Courts adopt this potentiality principle in their quest to define the term 'unborn' for the purposes of the Eighth Amendment/Article 40.3.3 of the Constitution, then it will still have to define when the embryo has the potential to become a human being.

Considering the contraveiling philosophical position holding that IVF embryos have no moral status that makes them worthy of protection, it has been argued that 'the most widely held view of the status of the embryo takes an intermediate position. It holds that the embryo deserves respect greater than that accorded to other human tissue, because of its potential to become a person and the symbolic meaning that carries for many people'[9]. This recognises that the embryo is a living entity deserving some respect, although not at the same level of protection as human persons. In other words, the IVF embryo before transfer should be afforded some protection due to its potential to develop into a human being and the symbolic significance it has [10]. Irish Courts might adopt this intermediate position when ascertaining the beginning of life in the context of a dispute over the fate of stored embryos.

### Regulatory dynamics in Ireland and the United Kingdom

Over the past 20 years, modern fertility treatments including IVF have become more widely available to medical consumers. Although the advanced reproductive technologies are now more culturally accepted than before, how these treatments are regulated in Ireland creates difficulty for both consumers and providers, if a couple has stored embryos and their personal or family situation changes. Given the persuasive authority of the UK legal system in Ireland, legislators in Ireland might consider the UK's legal response to assisted reproduction as they develop here. Regulation of IVF in the UK is based on the Human Fertilisation and Embryology (HFE) Act 1990 [11]. This Act established one organisation, the Human Fertilisation and Embryology Authority (HFEA), to supervise and license all fertility clinics throughout the UK It also provides information and advice to the British government about embryos and treatment services governed by the Act, and endeavours to ensure that the whole area of reproductive technology is practiced in a transparent manner. But because no similar unifying agency exists in Ireland, on matters of communication, control, and public accountability, the regulation of IVF in Britain is in stark contrast to that in Ireland.

Although regulation of the advanced reproductive technologies in Ireland is evolving, it remains fragmented and less sophisticated than in the UK The Medical Council was, by default, the only statutory body having any authority over IVF until recent years, when at least two other quasi-government bodies (the Irish Medicines Board and the Commission on Patient Safety and Quality Assurance) began to take an interest in regulating some aspects of assisted reproductive procedures.

Originally, the Medical Council exerted control over IVF because an agency with a specific remit for the advanced reproductive technologies was lacking. This circumstance necessitated IVF being regulated by the same board as all other branches of medicine. As of 2009, Ireland still does not have a single organisation mandated to coordinate monitoring and regulatory efforts for IVF, analogous to the HFEA in the UK Although the Institute of Obstetricians & Gynaecologists is the advisory body to the Medical Council on 'all matters relating to obstetrics and gynaecology in Ireland', when approached for direction or approval on providing specific IVF applications, the Institute issued a statement that it 'does not believe that it is within its remit to do so' [12].

The Medical Council was established under the Medical Practitioners Act [13] with responsibility for registering all physicians and setting professional practice standards. This body periodically publishes *A Guide to Ethical Conduct and Behaviour *[14] to provide 'a set of principles which doctors must apply in each situation, together with their judgement, experience, knowledge and skills'. The most recent Medical Council guidelines (March 2004) concerning IVF reminded doctors 'of their obligation to preserve life and to promote health. The creation of new forms of life for experimental purposes or the deliberate and intentional destruction of in vitro life already formed is professional misconduct' [14]. The Medical Council guidelines also state that IVF should only be used after thorough investigation has failed to reveal a treatable cause for infertility. Prior to fertilisation of an ovum, extensive discussion and counselling is essential. Any fertilised ovum must be used for normal implantation and must not be deliberately destroyed. Cryopreservation techniques have allowed greater flexibility in scheduling embryo transfers with no adverse effect on reproductive outcome, so IVF clinics can freeze surplus (non-transferred) embryos and remain compliant with Medical Council guidelines.

In response to EU Cell and Tissue Directive 2004/23/EC [15], the Irish Medicines Board (IMB) acquired a role in regulating IVF laboratories due to the designation of such facilities as 'tissue establishments'. The IMB exists to assure compliance with international standards regarding quality and safety for the donation, procurement, testing, processing, preservation, storage and distribution of human tissues and cells. The task of accreditation of all Irish IVF programmes was therefore taken up by the IMB with a view to develop a system for notification of adverse events and reactions, to organise inspections and control measures, as well as to ensure data protection and confidentiality. The IMB also focused on tissue traceability and record maintenance that could interface with a single European coding system.

In contrast to the broad nature of the Medical Council guidelines, the IMB approach to 'tissue establishments' is more narrowly defined. As 'tissue establishments', IVF providers in Ireland are required to secure a specific license from the IMB for human embryology laboratory operations. This IMB involvement in the arena of the advanced reproductive technologies, while perhaps appearing highly circumscribed, actually manifests a powerful regulatory presence over IVF since the embryology laboratory plays such a crucial role in the provision of IVF. Indeed, any IVF centre's laboratory licensure status as determined by the IMB also by extension determines the fate of that particular clinical IVF programme.

Yet another entity with regulatory input regarding assisted reproductive treatments is the Commission on Patient Safety and Quality Assurance (CPSQA). This commission was chartered to "develop clear and practical recommendations to ensure quality and safety of care for patients". It concluded its work in mid-2008, and proposed regular clinical audits and a three-year licensing scheme for IVF clinics as part of the 'Building a Culture of Patient Safety' initiative [16]. The government of Ireland also chartered a Commission on Assisted Human Reproduction (CAHR) to guide approaches for regulating the advanced reproductive technologies here [17]. The absence of any Irish legislation dealing specifically with assisted reproduction left the CAHR free to consider the question *ab initio*. This multi-disciplinary panel issued a comprehensive report in 2005 addressing social, ethical and legal factors to be taken into account in determining public policy [18]. As the most recent expert examination of reproductive treatments in Ireland, the CAHR report highlighted the fact that Medical Council guidelines alone have insufficient depth to ensure appropriate regulation of the advanced reproductive technologies. Having examined current legislation in the UK and other countries, the CAHR report concluded that regulation by statute was desirable in Ireland [18].

### Constitutional considerations in Ireland

According to the Eighth Amendment/Article 40.3.3 of The Irish Constitution [19], 'The State acknowledges the right to life of the unborn and, with due regard to the equal right to life of the mother, guarantees in its laws to respect, and, as far as practicable, by its laws to defend and vindicate that right.' It is unclear, however, whether the protection stipulated in the Eighth Amendment/Article 40.3.3 applies from the instant of fertilisation or from some subsequent point in the developmental process. In the modern age of IVF, this dilemma thrusts the most ancient of questions, 'when does life begin?', to the practical intersection of medicine and law [2]. It is not merely a philosophical exercise; indeed it must be posed in order to determine when Constitutional protections are afforded to the embryo [5,10,20]. When the question is settled, it will establish what might be done with the embryo and also who decides in the event of any dispute. The fact that this matter has not been addressed by legislative or judicial process has significant implications for patients seeking fertility treatment in Ireland. Clarification can only be obtained from a substantive pronouncement from the higher Courts or by way of Constitutional referendum.

This very controversy emerges in the matter of *Roche v. Roche et al *[21], a case certain to impact the future of fertility treatment in Ireland. The dispute involves a woman who wished to use embryos created by her eggs and her husband's sperm during their marriage in an attempt to become pregnant. After the couple had separated, the embryos remained in storage at an IVF centre in Dublin. Subsequently, her estranged husband refused to give consent for the embryos to be transferred. Mrs. Roche contends that the embryos had a 'right to life' by virtue of the Eighth Amendment/Article 40.3.3 of the Irish Constitution, and accordingly the embryos must be transferred in order to give them an opportunity to grow and develop in the womb and be born. The central challenge in the *Roche *case is establishing a definition of the term 'unborn' for the purposes of Constitutional law [21]. The vagueness of the term 'unborn' in Ireland is not new, and has been illustrated by legal difficulties in other contexts [22].

### Constitutional protection for the 'unborn' or 'non-transferred'?

An analysis of the campaign surrounding the 1983 Amendment to the Irish Constitution suggests that supporters of the amendment were satisfied that the term 'unborn' provided protection from the time of conception. The then-Deputy Prime Minister Richard Spring, said that 'It is clear that the word 'unborn' is likely to be interpreted by the Supreme Court as the moment at which the human ovum is penetrated by a sperm – the moment when human life commences' [23]. Yet the legally nebulous nature of the term 'unborn' makes it difficult to determine from which specific point in the fertilisation process the Constitution begins to protect life.

The case of *Attorney General (Society for the Protection of Unborn Children (Ireland) Ltd) v. Open Door Counselling Ltd *[24] concluded that the right to life of the unborn applied from the moment of conception. Indeed, the *Green Paper on Abortion *[25] concurred with the ruling that conception (fertilisation) is the starting point for statutory protection of the 'unborn'. But a close reading of the Court's decision raises the possibility that the event of fertilisation may have been erroneously equated with the related (but physiologically distinct) process of implantation, which, for IVF patients, is conditional upon undergoing embryo transfer. Indeed, it was asserted that the human embryo either *in vivo *(in the body) or *in vitro *(in the laboratory) is entitled to constitutional protection from the moment of conception [1,26-28]. If the embryo were to be guaranteed Constitutional protection from the instant of conception, then this could bring significant complications to the provision of reproductive medicine in Ireland. For example, if Constitutional protection were applied to all IVF embryos prior to *in utero *transfer, then would assisted embryo hatching (or any other beneficial/therapeutic procedure performed on an embryos) become assault? Certainly no action could be taken to allow IVF embryos to perish. Some authors [29] have expressed the view that 'it cannot be said with certainty whether the protection afforded by the Eighth Amendment/Article 40.3.3 to the "unborn" applies from the moment of fertilisation, the moment of the implantation or from some later date.' Nevertheless, in resolving a dispute over non-transferred embryos, Irish Courts may consider this judgment as it is the only substantive Irish precendent addressing the Constitutional status of the *'*unborn' to date.

*Attorney General v. X *[30] concerned an application by the State to prevent a fourteen year old girl (who was pregnant as a result of rape) from travelling to the UK for an abortion. There was evidence of 'real and substantial risk' that the girl would take her own life unless she left Ireland for the procedure. Mr. Justice Costello granted the injunction, but the Supreme Court reversed this decision on appeal and held that there was a real and substantial threat to the life of the girl, and that in such circumstances, the termination of pregnancy was constitutionally permissible [30].

Mr. Justice McCarthy added, 'I think it reasonable...to hold that the people when enacting the [Eighth Amendment to the Constitution] were entitled to believe that legislation would be introduced so as to regulate the manner in which the right to life of the unborn and the mother could be reconciled' [30]. This statement highlights the fact that, if the Court attempts to provide any definition of the 'unborn' in a dispute over the fate of non-transferred human embryos, then the wishes of the electorate who enacted the Eighth Amendment may require interpretation. Given the novelty of IVF in 1983, it will be difficult for the Court now to ascertain public sentiment now based on electoral data collected then. The Court may consider that if the electorate were aware of the various contingencies associated with the advanced reproductive technologies, then they would have perhaps wanted the embryo to be protected from the earliest time possible, *i.e*. from the moment of fertilisation.

In a dissenting opinion [30], Mr. Justice Hederman commented that distinctions could not be made between the phases of unborn life before birth. It was his position that Constitutional protection was not restricted by any condition the unborn was required to attain: 'The Eighth Amendment establishes beyond any dispute that the Constitutional guarantee of the vindication and protection of life is not qualified by the condition that the life must be one which has achieved an independent existence after birth. The right of life is guaranteed to every life born or unborn. One cannot make distinctions between individual phases of the unborn life before birth or between unborn and born life' [30].

This case generated intense debate throughout Ireland regarding the socially charged issue of abortion, and highlighted the inconsistencies in the Constitutional provison governing the protection of the 'unborn' [31]. The Constitutional Review Group (CRG) [32] subsequently focused on this point and recognised that the lack of clarity surrounding the definition of the unborn was creating significant legal and moral difficulties: 'There is no definition of "unborn" which, used as a noun, is at least odd. One would expect "unborn human" or "unborn human being". Presumably the term "unborn child" was not chosen because of the uncertainty as to when a foetus might be properly so described. Definition is needed as to when the "unborn" acquires the protection of the law. Philosophers and scientists may continue to debate when human life begins, but the law must define what it intends to protect. "Unborn" seems to imply "on the way to being born" or "capable of being born". Whether this condition is obtained from fertilisation of the ovum, implantation of the fertilised ovum in the womb, or some other point has not yet been defined' [32].

The expression 'on the way to being born', as recommended by the CRG, supports the argument that the non-transferred embryo should not be guaranteed protection by the Eighth Amendment/Article 40.3.3 of the Constitution. This is because without transfer and subsequent implantation in the uterus, the embryo cannot be assured to be ' on the way to being born' or 'capable of being born'. Indeed, many fertilised eggs are lost naturally and never implant in the uterus. As the All-Party Oireachtas Committee on the Constitution in its Fifth Progress Report on Abortion stated: 'great numbers of fertilised ova are lost in the natural course of things and never become implanted in the uterine wall. As a result some argue that implantation is the decisive event in the development of unborn life' [33].

An alternate interpretation offered in the CAHR report suggested that new human life exists once the process of fertilisation is complete; the embryo is more than simply a cluster of cells – for once destroyed, it is impossible to recreate that particular life [34]. From this position, it seems evident that any court decision concluding that a non-transferred embryo from IVF has the same 'right to life' as the embryo formed without assistance would have a profound effect on the provision of IVF in Ireland. Such a definition would certainly have implications for any couple engaged in litigation over the fate of stored embryos, since the non-transferred human embryo would legally acquire the classification as an 'unborn' for the purposes of the Eighth Amendment/Article 40.3.3. By extension, if such a definition were to be legally instituted, in order to 'defend and vindicate' its 'right to life' the State would seem obliged to have some legal mechanism to *require *the embryo to be transferred to a uterus. But would this level of governmental involvement override the procreational right of any party wishing to avoid parenthood [35,36]? This brings forward the important issues of 'abandoned' embryos [37] currently in storage at Irish fertility clinics, and the equally vexing challenge of mandatory transfer of embryos known to carry a genetic disease [38]. By extension, Irish Courts might determine that all IVF embryos come within the protection of the Eighth Amendment/Article 40.3.3. If this were to be the case, it would directly impact IVF providers and patients since it would require that all embryos produced would have to be transferred to the woman's uterus. Presumably, this would have to include the transfer of embryos even if they were found to be defective via pre-implantation genetic diagnosis. However, if the Eighth Amendment/Article 40.3.3 were determined *not *to apply to non-transferred IVF embryos, then there would be no Constitutional impediment to pre-implantation genetic diagnosis where embryos are "de-selected" for transfer based on a genetic condition. In 2005, the CAHR report recommended that the embryo formed by IVF should not attract legal protection until it was transferred *in utero*, at which stage it should have the same legal protection as the embryo created *in vivo *[18].

But is procreative liberty (the personal freedom either to have children or to avoid having them) Constitutionally protected? If it exists at all, any legal 'right' to reproduce appears to be a negative right, meaning that other persons have a duty not to interfere with the exercise of procreative choice. It does not imply any obligation for others to provide resources necessary to exercise that choice [39].

In *Hecht v. Superior Court of Los Angeles County *[40], the applicants wished to have cryopreserved sperm destroyed 'to prevent the birth of a fatherless child, disruption of their existing family, and additional emotional, psychological and financial stress'. In a dispute over the fate of frozen IVF embryos, a similar argument may be advanced by the party seeking to avoid procreation. In the *Hecht *case, the Court emphasised the argument that the embryo constituted property by ruling that Hecht was entitled to use the sperm bequeathed to her.

Where a conflict of procreative autonomy has arisen, thus far judges tend to favour the party seeking to avoid procreation, as the alternate decision would force unwanted parenthood on that party [35,41,42]. The Irish judiciary could adopt a similar approach should any conflict of procreative rights arise here. Undoubtedly, the relevance of the Constitution to such rights will also have to be considered by the court.

In a dispute over frozen embryos, the party wishing to use the embryos for transfer may argue that they have a Constitutional right to procreate, and any legal manoeuvre to prevent embryo transfer would be an infringement of that right. This argument may be forwarded if the court decides that the embryo is not protected by virtue of the 'right to life' guarantee contained in the Eighth Amendment/Article 40.3.3 of the Irish Constitution. Indeed, this may also be argued if the embryo is regarded as having an intrinsic 'right to life', as procreational rights will then be assessed without the impingement of constitutional provisions. However, while there is a Constitutional right to procreate, that right is not absolute in its guarantee. Article 40.3.1 protects a host of unenumerated rights, one of which is the right to procreate.

This issue first came before the Court in *Murray v. Ireland *[43], where the plaintiffs were husband and wife both serving sentences of penal servitude for life. They had no children and conjugal visits were denied during incarceration. The plaintiffs claimed that as a married couple, they had a right to beget children, that this right was protected by the Irish Constitution, and that it was being infringed as a function of their imprisonment. The plaintiffs argued that as lawfully married persons, they had a basic human right to procreate and that this right was Constitutionally protected. Although they argued that there was a hierarchy of protected rights, and that the right to procreate children was very high on that scale of values, the Court rejected this claim [43]. Such a position may be taken by a party seeking to avoid procreation where the non-transferred embryo has been accorded Constitutional protection.

### A legislative approach for Ireland

Considering the absence of a clear Constitutional role in the regulation of assisted reproduction in Ireland, political leaders may be influenced by legislation in the United Kingdom. The issues of greatest concern were addressed by the HFE Act 1990. In response to an amendment to this Bill seeking to clarify the legal status of the human embryo, a former Lord Chancellor stated, 'An embryo is not a chattelA human entity which is living is not a chattel and neither is it a person in any ordinary senseIt is wrong to try and define a human embryo in terms of established legal definitions which are plainly inapplicable to human embryos. Why must an embryo be one or the other? Why cannot it just be an embryo?' [44].

These remarks from the UK suggest that the non-transferred embryo is *sui generis*, occupying a category of interim property. In any event, the amendment was withdrawn and consequently the legal status of the embryo remained undefined. The Warnock Report [8] which preceded the HFEA, expressed that 'Until now the law has never had to consider the existence of embryos outside the mother's uterus. The existence of such embryos raises potentially difficult problems as to ownership. The concept of ownership of human embryos seems to us to be undesirable. We recommend that legislation be enacted to ensure that there is no right of ownership in a human embryo' [45]. But creating a new, third category of 'interim property' may not fully remedy the challenges brought by non-transferred IVF embryos. It has been claimed that 'the court would have no choice but to treat an extra-corporeal embryo as either a person or a chattel. The likely outcome would be that it would be held to be a chattel. Such law that exists points in this direction and the pragmatism of the common law would see that to treat an extra-corporeal embryo as a chattel is more consistent with common sense than for it to be given the rights of a person' [46].

While the HFE Act 1990 does not explicitly define the legal status of the embryo, it does vest control of such embryos in the providers of the genetic material [11]. In the UK, this is accomplished by a mechanism whereby the consents of gamete providers determine the disposition of the embryos and define the power to control the use of the embryos derived therefrom. Specifically, the gamete provider must, at the time that the gametes are procured, indicate in writing for what purpose(s) those gametes may be used. The gametes, and/or any resulting embryos may only be used in accordance with the provisions obtained from written informed consent [36,47,48]. In the UK, this documentation confers legal control to the gamete providers, thereby minimising the type of disputes which have taken place in the United States regarding the disposition of gametes and embryos. The IVF provider must provide thorough counselling and obtain an effective written informed consent from the couple seeking treatment (the gamete providers) to delineate the scope of use to which the gametes and/or embryos may be put [49]. While the topics covered in these medical consents are unpleasant and may be awkward for couples about to undergo IVF, it is imperative that the treating clinic makes an effort to document the patients' wishes across a wide range of contingencies before commencing the IVF sequence.

These scenarios should include what is to be done with gametes and/or non-transferred embryos if the couple separate or divorce, if the gamete donor dies or becomes incompetent, the use to which the material can be put, and the maximum period for which the material can be cryopreserved. The task of discussing these various inter- and post-treatment eventualities is assigned to the physician who collects the informed consent, because the IVF doctor is considered to be the individual best suited to respond to medical questions arising from the advanced reproductive technologies. A similar regulatory process could be introduced in Ireland to combat the inconsistencies that emanate from the absence of explicit regulation.

Indeed, it has been suggested that an IVF informed consent defining the disposition of non-transferred embryos would do much to avoid future unspecified situtations that may come before Irish courts [46]. The legislative model in the UK gives dispositional control to the gametic contributors of the embryo via a detailed written informed consent process that must be obtained before commencing IVF treatment. While Irish legislators could adopt a similar approach, they will have to be mindful of any determinations the Courts assert in the current *Roche *case regarding the constitutional status of such embryos.

### A property model for non-transferred embryos?

Judges and legislators must now regard embryos and gametes in ways never before necessary or possible. If Irish courts do not ascertain that the frozen IVF embryo deserves Constitutional protection, then they could rely on the property model as a determining factor in resolving a dispute over the fate of frozen embryos. Such 'a property analysis may allow more flexibility in defining the legal status of the embryo and properly serve the needs of public policy' [36,47,50].

The lack of legal authority in Ireland has significant implications for the landmark *Roche *case now before the Irish judiciary. In looking to other jurisdictions, the Court may explore the issue of whether or not the embryo can be perceived as property for the purposes of ascertaining who has dispositional control over that embryo. However, this approach is not without difficulty: 'In law it is usual to categorise things into property, which can be owned and controlled, and persons, which cannot. It is difficult to decide which of these categories is most appropriate for the embryo' [39].

The classification of a non-transferred IVF embryo as property means that its ownership will be vested in the parents of those gametes that gave rise to the embryo. This would permit the couple to determine and control the fate of their IVF embryos including disposal of any such embryo(s) in any way they wished.

Yet, there is the proposition that the language of property is inappropriate for discussions of the human embryo. Indeed, it may be argued that it is unrealistic to suggest that one person has 'property' in another whole living body. A host of complex legal issues also would emerge from this application of the property model in assisted reproduction including which of the parents can claim custody, the possibility of sharing embryos equally between the disputing couple, and the consequent moral difficulties that follow. Because this issue has come before senior courts in other jurisdictions, Irish judges may consider the settled case law elsewhere to ascertain how best to resolve a dispute over the fate of stored embryos here.

In the United States, a property model was applied in *Davis v. Davis *[51], a case regarding non-transferred embryos. In this dispute, the central question was who should have dispositional control over stored IVF embryos following divorce. The ex-wife wished to use the embryos in an attempt to become pregnant. The ex-husband did not wish to become a father, and further contended that to proceed with embryo transfer against his wishes was a direct infringement of his right to avoid procreation. The Tennessee Supreme Court considered the 'person v. property' dichotomy, and held that 'Pre-embryos are not, strictly speaking, either 'persons' or 'property' but occupy an interim category that entitles them to special respect because of their potential for human life'. Judgment was affirmed in the ex-husband's favour.

Therefore, although the Tennessee Supreme Court rejected the application of an absolute property model in *Davis*, it did recognise the decision-making control over the embryo by the couple. That decision was influenced by a report by The American Fertility Society, which recommended that 'Within the limits set by institutional policies, decision-making authority regarding pre-embryos should reside with the persons who have provided the gametes...in the absence of specific legislation on the subject' [52]. However, the matter of decisional authority has been criticised as just another way to ask the basic question of who 'owns' the IVF embryo. As one author noted 'Ownership does not signify that embryos may be treated in all respects like other property. Rather the term merely designates who decides which legally available options will occur, such as creation, freezing, discard, donation, use in research and placement in a uterus. Although the bundle of property rights attached to one's ownership of an embryo may be more circumscribed than for other things, it is an ownership or property interest nonetheless' [53]. With respect to the property model, the decision in *Davis *could be regarded as an instance where 'recognition of the existence of a proprietary or perhaps pseudo-proprietary or quasi-ownership interest' [54] prevailed. Accordingly, if the IVF embryo constitutes property and belongs to the couple who contributed the genetic material to create that embryo, then it follows that the couple should have decisional authority over its fate.

The property model was also applied in *York v. Jones *[55], where an IVF clinic refused to transport embryos to California despite a request from the couple involved. The Court considered the bailor-bailee relationship that existed between the couple whose non-transferred embryo from IVF was stored in a fertility clinic [54]. The Virginia clinic argued that their contract with the couple only allowed for three possible dispositions in the event the couple did not proceed with IVF at the clinic of origin: 1) embryo donation to another infertile couple, 2) donation for human embryo research, or 3) destruction. The *York *decision is significant because the language used is an explicit recognition of the couple's proprietary interests in the embryo. The case was decided in favour of the couple, as the Court noted that the medical treatment agreement did not state that the attempt to achieve a pregnancy was limited to procedures employed at the clinic of origin.

*Paraplaix v. CECOS *[56] also framed the issue of reproductive autonomy in a property context. This matter concerned the use of sperm for posthumous reproduction, where a widow requested the release of sperm from her deceased husband in order to achieve pregnancy posthumously. However, he had not made any specific disposition of the sperm his will. His widow nevertheless contended that the sperm formed part of the property of the deceased's estate, and was thus capable of being inherited. In contrast, CECOS argued that sperm was an indivisible part of the decedent's body (similar to a limb) and was therefore not inheritable as property. The widow was ultimately successful on grounds of her procreational autonomy argument, but not on the basis of the property argument. The Court held that 'sperm does not constitute a thing in commerce but secretion containing the seed of life destined for human procreation'.

While *Paraplaix *involved gametes rather than IVF embryos, it remains relevant because it offers one route Irish courts might take in construing the embryo as property. Since this decision held that 'sperm does not constitute a thing in commerce', if the embryo is aligned with sperm for analysis purposes, then Irish Courts might also reject the property model. The parallel is imperfect, however, since it could be argued that someone has a right to decide what is to be done with stored sperm. The person who made the deposit should 'own' or have a property interest in the stored sperm as it 'is his semen in a biological and property sense, and thus he has the right to decide what happens to it' [53]. Furthermore, sperm is significantly different from the non-transferred embryo as the latter is created by sperm and ova derived from a participating couple, unlike sperm which is the simple deposit of one individual – a specimen by itself lacking the potential to develop into a human being. For frozen sperm, a property analysis can therefore be more easily argued.

Although these precedents in other jurisdictions could influence Irish Courts in determining which model to adopt where the fate of surplus embryos created by IVF is disputed, this influence hinges on the applicability of the Constitution to the IVF embryo. Should Irish courts determine that Constitutional protections are not applicable to non-transferred embryos, then a modified property model may be used. This does not accept the property approach absolutely, but rather would recognise the respect owed to the human embryo while acknowledging that the participating couple have an interest in what happens to the subsequent embryo.

### But is the non-transferred embryo ever 'owned'?

To ascertain if the embryo can be regarded as 'property' so as to allow one party in a dispute to exercise control over the destiny of that embryo, it is useful to analyse the debate surrounding the ownership of other body materials or components. Intuitively, it is quite natural for an individual to think in terms of 'my body' and to infer that because it is 'my' body, that the person can determine precisely what is done to it, or its parts [57]. In *Anarchy, State and Utopia *[58], the concept of 'self-ownership' was introduced and has since figured prominently in philosophical debates on issues ranging from taxation and exploitation to suicide, abortion and surrogacy [59]. 'Since there is an open-ended set of use-privileges and control-powers over one's own body, it seems natural enough to speak of 'owning' one's bodyif I am not a slave, nobody else owns my body. Therefore I must own myself. Therefore I must own all my actions, including those which create or improve resources. Therefore I own the resources, or the improvements, I produce' [60].

The matter of body ownership was explored in *Doodeward v. Spence *[61], which highlighted a dispute where the plaintiff sought the return of the preserved corpse of a two-headed stillborn baby from the police. The plaintiff claimed that he had a 'property' claim over the corpse. The Australian High Court agreed, stating 'when a person has by the lawful exercise of work or skill so dealt with a human body or part of a human body in his lawful possession that it has acquired some attributes differentiating it from a mere corpse awaiting burial, he acquires a right to retain possession of it, at least as against any person not entitled to have it delivered to him for the purpose of burial, but subject of course, to any positive law which forbids its retention under the particular circumstances.'

The Court of Appeal in *R v. Kelly & Lindsay *[62] accepted the methodology of *Doodeward*, and concluded that in certain circumstances parts of corpses are 'property' and are therefore capable of being stolen. In *Kelly*, a sculptor was alleged to have stolen human body parts from the Royal College of Surgeons. The question that arose for the Court was: Could these body parts be considered as property in order to convict the accused of theft? The Court held that parts of a dead body may be property (and may therefore be capable of being stolen) when 'they have acquired different attributes by virtue of the application of skill, such as dissection or preservation techniques, for exhibition or teaching purposes'. This exception, coupled with the decision in *Doodeward*, may be useful to Irish Courts in drawing conclusions that the embryo may be considered to constitute property.

Application of the principles from *Doodeward *may yield a useful analogy to IVF embryos, as their creation certainly requires an element of special skill. It should also be noted that the storing and thawing of embryos presents some technical challenge, and could be regarded as constituting 'preservation' for the purposes of affinity with *Doodeward *and ascribing a property status to the IVF embryo.

Interesting and complicated questions will arise as ownership rights over non-transferred IVF embryos are explored in Ireland. Perhaps the State could recognise property rights for IVF embryos, but also devise a framework within which those rights must be exercised. Otherwise, there could be some judicial hesitancy to resolve a dispute over stored embryos in the context of a pure property model. Furthermore, in the absence of a legally defined framework, application of an absolute property model to non-transferred embryos could provide a couple with dispositional control the power to excericse unlimited rights over their embryos – potentially doing whatever they wished with that 'property'.

### Informed consent and non-transferred embryos: limitations and legal challenges

While the legal status of non-transferred embryos awaits legal and/or judicial clarification, the process of informed consent still must be transacted between IVF patients and their doctors. Medical informed consent is a crucial factor in any dispute arising over the disposition of stored embryos. In a medical context, informed consent is a formal process by which a patient can be said to have given permission for a proposed treatment based on a full and clear appreciation and understanding of facts, the implications and future consequences of the proposed treatment, the expected outcome(s) following treatment, awareness of alternative treatment(s), and the possible consequences of refusing treatment. To give informed consent, the individual concerned must have adequate reasoning faculties and be in possession of all relevant facts when consent is given. Unless a valid informed consent is obtained, any subsequent physical interaction between doctor and patient constitutes assault, unless the encounter is covered by the concept of necessity. Since IVF is always an elective medical procedure, therapy cannot begin unless the treating physician obtains written informed consent first [36,47,63].

Although there have only been a small number of cases involving the status of IVF embryos thus far, some Courts have regarded the informed consent (when properly executed) as a binding contract, and decide who shall have dispositional control over the embryos on that basis [20,47]. In an Irish context, if Courts should adopt this contractual model in order to decide 'custody' of any disputed non-transferred embryos, then this would allow the judiciary to avoid the difficult questions raised by the Constitutional status of the 'unborn'. Unfortunately, the language of informed consent for IVF in Ireland is not uniform and has never been standardised, so variation among clinics could be problematic.

As background, surplus or non-transferred embryos from IVF may be safely cryopreserved for extended intervals [64,65], although a specific informed consent is also required for this procedure. In Ireland, there is no limit to the duration of such storage, in contrast to the UK, where embryos are frozen for a maximum period of ten years. After ten years, surplus embyos in the UK are thawed without transfer, and thus are 'allowed to perish'. It should be noted that the 'thaw without transfer' provision for non-transferred embryos permitted in the UK cannot be easily adopted in Ireland, due Medical Council guidelines and the unresolved question of Constitutional protection of the unborn as contained in Article 40.3.3.

The potential conflict between 'thaw without transfer' and the Eighth Amendment/Article 40.3.3 of the Irish Constitution notwithstanding, some IVF providers in Ireland include language in the written informed consent stipulating certain circumstances where the clinic retains the right to dispose of non-transferred embryos by thawing them without transfer. Patients undergoing an IVF sequence at such a facility must agree in writing that when the wife's age exceeds 45 years, or when the cryopreserved embryo(s) have been in frozen storage in excess of 5 years – whichever occurs first – any non-transferred embryos will be removed from storage, thawed, but not transferred (*i.e*. destroyed).

Any informed consent executed in Ireland containing such a 'thaw without transfer' clause invites the possibility of a judicial test of contract enforcability. This is because institutions using this type of informed consent in Ireland make access to IVF conditional upon a couple's agreement that their non-transferred embryos could be 'thawed without transfer', a procedure itself inconsistent with current Medical Council guidelines. While this may have been influenced by standards lawfully set in the UK, a written informed consent in Ireland enumerating situations where intentional destruction of human embryos by 'thaw without transfer' treads on terrain that has not yet been Constitutionally tested.

In the event of circumstances changing in the relationship of the couple who underwent IVF treatment, such as the death of either partner, separation or divorce [63,66], what would be the fate of any non-transferred cryopreserved embryos? What should be the scope of informed consent for IVF in such cases? From a public policy perspective, is an IVF clinic's registration status jepordised (with the Medical Council) if it incorporates a standard 'thaw without transfer' clause into its treatment agreement? Would such an informed consent be considered legally enforcable? How might Irish Courts analyse this issue? The Irish Constitution (particularly Article 40, regarding the 'unborn' and personal rights) is the only substantive law in Ireland which can be applied to these matters at present.

Courts in Ireland may consider decisions in related cases from other jurisdictions to resolve such issues. The UK case, *R v. Human Fertilisation and Embryology Authority (ex parte Blood) *[67] involved a dispute over the fate of surplus embryos and specifically addressed informed consent. Under existing UK law the HFEA was allowed to prohibit a woman from using her husband's sperm in the UK, because it was obtained without consent from her husband (who was incapacitated due to a comatose state). In 1997, the HFEA permitted the wife to export the sperm to Brussels for use in an IVF treatment there. The Court of Appeal's judgement confirmed that the principle of informed written consent is an essential part of English law, and that the posthumous storage or use of sperm or eggs without such consent is unlawful.

The European Convention for the Protection of Human Rights and Fundamental Freedoms (ECHR) [68], offers a useful appeal mechanism for any individual from the E.U. wishing to revisit a Supreme Court decision regarding the status of non-transferred embryos. In Article 12, this convention provides that 'Men and women of marriageable age have the right to marry and to found a family, according to the national laws governing the exercise of this right.' And, since Article 8 states that 'Everyone has the right to respect for his private and family life' [68], it could be argued that the right to procreate is inherent in either ECHR article. Accordingly, should the Supreme Court of Ireland make any pronouncement on the Constitutional right to procreate, then the *Roche *case could be taken before the European Courts with the assertion that the decision infringed upon the individual right to procreate (or to avoid procreation) under the ECHR. That Court's judgment is binding on each EU member state that has ratified the Convention, however there is no mechanism by which the member state can be forced to implement that judgment. Unlike EU law, the ECHR cannot overrule the Constitution of Ireland, although many of the rights in the ECHR are already contained in the Constitution of Ireland. Thus, although individuals have the opportunity to seek appeal in the European Courts, it is evident that the State may avoid any obligation to implement a judgment from European Courts if such judgment is inconsistent with the Irish Constitution.

An example of this chain of appeal of a member state's Supreme Court to the European Court is *Evans v. Johnston HFEA et al *[69]. This case presented the first opportunity the UK had to address the question of who should decide the fate of a frozen embryo when disputed by its two progenitors [70]. Following passage through the highest levels of the English judicial system, the *Evans *case was deliberated by the European Court of Human Rights. Due to the lack of precedent regarding the fate and status of non-transferred embryos in Ireland, the substance and trajectory of the *Evans *case are likely to affect the *Roche *case, or any similar case, brought before Irish Courts.

In this dispute, Ms. Evans and Mr. Johnston were engaged to be married and the couple had undergone IVF treatment as chemotherapy treatment left Ms. Evans infertile. They had pre-implantation embryos in storage intended for future use. For Ms. Evans, the frozen non-transferred embryos therefore represented her only chance of having a child to which she was biologically related. When the relationship ended and Mr. Johnston decided not to proceed with IVF, it was contended that his withdrawal of consent would interfere and permanently frustrate Ms. Evans' overwhelming ambition to have children. The central issue here was whether the interference with her private life was necessary and proportionate given the circumstances. Ms. Evans advanced an number of arguments that the English law's interference with her private life was unlawful. Mr. Johnston wanted the six non-transferred embryos destroyed, while Ms. Evans wanted to use them to achieve pregnancy.

Ms. Evans sought an injunction from the English courts compelling Mr. Johnston to restore his consent. She claimed that: (i) Mr. Johnston may not vary or withdraw his consent to the use of the embryos, (ii) The embryos may be stored throughout the remainder of the ten year period, (iii) Ms. Evans may lawfully be treated with embryos during the storage period, (iv) A declaration of incompatibility to the effect that Section 12 and Schedule 3 of the 1990 HFE Act 1990 breached her Article 8,12 and 14 rights of the ECHR, and (v) That the embryos are entitled to protection under Articles 2 and 8 of the ECHR [70].

In UK Courts, Ms. Evans unsuccessfully argued that her ex-partner should not be allowed to withdraw his earlier consent to the creation, storage, and use of the embryos. The Court took notice that under the HFE Act 1990, consent from both partners is required at *every stage *of the IVF process, including transfer. The Court of Appeal dismissed her appeal and held that Ms. Evans and Mr. Johnston only gave consent to the use of the embryos for 'treatment together'. As the couple were no longer together, the informed consent provided by Mr. Johnston's at the beginning of the IVF sequence was considered no longer valid. The Court highlighted that under Schedule 3 of the HFE Act 1990, Mr Johnston had an unconditional statutory right to withdraw or vary consent to the continued frozen storage of the embryos as well as their transfer. The Court of Appeal also held that the statutory requirement of continuing consent did not breach the European Convention on Human Rights, as it was proportionate to the legislative aim of protecting the rights and freedoms of both parties. The Court stated that a non-transferred embryo does not have a qualified right to life under Article 2 of the ECHR. While the Court of Appeal determined that the refusal of treatment (due to Mr Johnston's withdrawal of consent) was an interference with Ms. Evans' right to respect for private life under Article 8 of the ECHR, the Court held this interference was proportionate to the need that made it legitimate. They did not recognise that this was Ms Evans's last chance to have a genetic child [70].

After exhausting all avenues of appeal in the UK, Ms. Evans next turned to the European Court of Human Rights in Strasbourg, arguing that by making the exercise of her reproductive rights dependant on Mr Johnston's consent, her rights governed by Article 8 had been infringed. The European Court found that such a position would diminish the respect owed to Mr. Johnston's private life in proportion, as it enhanced the respect accorded to hers. The Court also noted that a balance must be struck between Ms. Evans and Mr. Johnston's privacy rights, yet the legislature (acting through its designee, the HFEA) had done this by requiring consent from both parties. Ms. Evans also argued discrimination under ECHR Article 14, on the grounds that in natural reproduction, men cannot withdraw consent to reproduction once the sperm has been voluntarily given whereas in IVF, men could modify or withdraw their consent at various stages. Nevertheless, the European Court decided that this difference in IVF was justifiable because even though in natural conception the male does not have the right to withdraw consent, the HFE Act 1990 aimed to treat both parties equally even though this does not happen in nature. The UK scheme of consents inherent in the Human Fertilisation and Embryology Act was therefore upheld, with Mr. Johnston prevailing [69].

It should be noted that Ms. Evans' argument that the non-transferred embryos had a 'right to life' under ECHR Article 2 was denied. The Court denied this argument because English law does not protect the rights and interests of the foetus prior to birth thus, so it would be anomalous to give such rights to non-transferred embryos. However, application of this component of the *Evans *decision in Irish law will be difficult given the differences between Ireland and the UK with respect to Constitutional issues.

*Evans *was the first case on fertility treatment to be considered by the Human Rights Court, and the judges acknowledged that there was no international consensus about its regulation or to the status of non-transferred embryos. The Court was sensitive to the fact that their verdict deprived Ms. Evans of the ability to give birth to her own child, but they did not seem to consider that this may be the determining factor to allow her use of the stored embryos. The Court also insisted that Ms. Evans's inability to override the withdrawal of consent by the other 'genetic parent' did not breach her human rights.

While some commentators on the *Evans *case recognised that women's investment in pregnancy, child-birth and the IVF process is greater than men's [42,71], no jurisdiction thus far has regarded this as a sufficient reason to elevate female rights above those of the male, with respect to ECHR Article 8. Given the lack of precedent in Ireland, the *Evans *case is likely to be a substantial persuasive authority on the *Roche *case, or any similar case brought before Irish Courts.

*AZ v. BZ *[72] furnishes another example where the validity of consent forms signed by a couple before beginning IVF treatment and embryo storage was considered. The Court held that a consent paper signed by a man stipulating that in the event of separation his wife could have the use of stored embryos, could not be enforced against him years later, as it would require him to become a parent against his wishes. The Court further held that agreements entered into by the couple when they initiated IVF should be enforceable, but determined that either party could later change his or her mind about the disposition of the embryos, and if, after a divorce the parties disagreed as to disposition of any non-transferred embryos derived from IVF, then the party wishing to avoid procreation should prevail. The Appellate Division of the New Jersey Superior Court held that 'a contract to procreate is contrary to New Jersey public policy and is unenforceable', relying on the 1988 decision *In the Matter of Baby M *[73], which held that a maternal surrogacy contract was unenforceable as a matter of public policy. In New Jersey, the Court ultimately decided that the right not to procreate outweighed the right to procreate. While the Court suggested that it was willing to enforce agreements dealing with non-transferred IVF embryos, its rule ultimately rendered all such contracts unenforceable. This is because mere disagreement by either party vitiates the contract in favour of the non-procreative rights of the party wishing to disallow embryo transfer and subsequent potential pregnancy.

A thoroughly prepared and properly obtained written informed consent prior to IVF can still be helpful at law [48,63,66]. The verdict in *Roman v. Roman *[74] determined that a written informed consent signed by a couple specifying that their frozen embryos be discarded in the event of divorce was valid. The ruling reversed a trial Court's award of frozen embryos to the wife, who had wanted to use them for *in utero *transfer and potential procreation. The Court found that written agreements between embryo donors and fertility clinics to which all parties have consented are valid and enforceable, provided that the parties have an opportunity to withdraw their consent to the terms of the agreement. The Court determined that the public policy of Texas would permit a husband and wife to enter voluntarily into an agreement before IVF treatment commences and make provisions for the disposition of any non-transferred embryos in the event of contingencies such as divorce, death or changed circumstances. The decision in *Roman *suggests that carefully drafted IVF consent forms can be enforced, consistent with public policy.

Indeed, the preliminary ruling in the *Roche *case directly addressed the issue of informed consent, and hinged on whether Mr. Roche had given consent for the future implantation of any non-transferred embryos [75]. Mrs. Roche asserted that her former husband was the legal father of the frozen embryos, having signed a contract agreeeing to take full responsibility for the outcome of IVF. But Mr. Roche told the Court that circumstances had changed, he no longer wanted to have more children with his former wife, and claimed that they never made an agreement about the future use of frozen embryos [76]. The Court ruled that Mr. Roche had not given his express or implied consent for future utilisation of the non-transferred embryos.

Regarding this case, an officer of the Irish Fertility Society stated that, 'Each episode of treatment requires the consent of all parties involved and this consent may be revoked by any party should their circumstances changeWe cannot accept that either partner should be coerced into any fertility treatment, even if he or she has already had treatment which has lead to the creation of embryos' [21].

Yet is there merit in an alternate view that assumes the couple, by agreeing to undergo an elective medcial procedure such as IVF, create an implied contract to use any embryos derived from such treatment to attempt to have a child? Any subsequent disputes should therefore be resolved in favour of the party seeking to procreate, as that was the original intention of the treatment [77-79]. Voluntary participation in the IVF process could be regarded as conduct reasonably leading to the assumption that both parties have committed to reproduction. In the event of changed circumstances, the doctrine of promissory estoppel becomes essential, as the party who subsequently seeks to use any non-transferred embryos relies, to his or her detriment, on the other party's commitment to reproduce jointly: 'The partner who opposes implantation of the embryos should be estopped from asserting his or her right not to reproduce'[80].

But the Court determined that when Mrs. Roche initially gave informed consent when she agreed that the embryos would be transferred *in utero*, that the three embryos mentioned in the informed consent document were those which were transferred 'fresh', not frozen. The purpose of cryopreservation of any surplus embryos was to use them if the first 'fresh' transfer failed. Mr Justice McGovern noted that, 'In light of the evidence in this case and the documents which have been produced, it cannot be said that it was the presumed intention of the parties that the three frozen embryos would be implanted in the woman's uterus in the circumstances which have arisen, namely following the success of the first implantation procedure and the legal separation of the couple' [76].

In *Litowitz v. Litowitz *[81] the written informed consent stipulated that any non-transferred embryos be allowed to perish after five years in storage. A dispute developed after the couple separated, as the husband wanted to put the embryos up for adoption and the wife wanted to have them transferred to a surrogate mother. In this case, there was also an egg donor involved who wanted the embryos back if the wife did not prevail. The Court considered the husband's interest to avoid procreation as stronger than that of the wife, and ruled in his favour.

While parties can trust courts to 'do the right thing' by balancing the conflicting interests should disputes over non-transferred embryos arise, IVF clinics have a responsibility to craft their informed consent in a way that is as comprehensive as possible [47,63]. This is because medical practitioners have the best expertise in the field and are keenly aware of the potential difficulties that could develop [82]. It was just such an unfortunate outcome that led to the dispute in the *Roche *case, where 'neither the wife or the husband adverted to the issue [of embryo disposition] until their marriage broke downin the absence of the consent forms indicating agreement either expressly or by implication, there was no agreement as to what was to happen to the three frozen embryos in the circumstances which have arisen' [75]. One of the curious features about the *Roche *case was that the consent forms provided at the time treatment commenced did not specifically address the disposition of any frozen embryos [83], either in the event that pregnancy was achieved from the first embryo transfer, or if their circumstances changed such as upon the death of either partner or a separation or divorce. The informed consent process in the *Roche *case was therefore found unsatisfactory from a legal sense [76], because it outlined the fresh transfer sequence but did not anticipate contingencies arising from any cryopreserved embryos. Informed consents for IVF in Ireland mandating 'thaw without transfer' are at risk of similar judicial criticism, but for different reasons. While the Court's decision in the *Roche *case provided some minimal conditions for how informed consent should be structured for IVF in Ireland, the fertility clinic that treated the Roche's had already modified their consent process before the case became public. Nevertheless, clarification about what should and should not be included in Irish IVF consent forms is needed.

## Conclusion

The two conflicting interests in cases dealing with the disposition of non-transferred embryos represent the right to procreate, and the right to avoid procreation [2,20]. The obvious problem for Irish Courts, in common with all other jurisdictions, is that enforcement of one person's rights denies the rights of the opposite party [84,85]. It is therefore necessary to determine whether these rights are equal, or whether one right should outrank the other [86].

The 'right to procreate' has been variously regarded as either superior to the 'right to avoid procreation', or that the 'right to avoid procreation' does not exist at all in the context of IVF [63,85]. Similarly, it has been argued that there is only a negative moral right to have children, therefore no one should be allowed to interfere with procreative efforts. Couples have no positive moral right to have children, which implies that childless couples have no positive right to assisted procreation and that the State has no obligation to provide such services.

While various arguments posit that the rights of the party wishing to avoid procreation from use of non-transferred embryos should prevail [87], an exception has been suggested for the partner who has no other possibility of having a genetic child except by transfer of such embryos [79]. It has even been suggested that 'if the non-consenting party simply wants to avoid having custody or financial reponsibility, a court could convert the party's status from being the parent of a frozen embryo to being an "egg donor" or "sperm donor" without the custody or financial obligations of parenthood' [88]. But whether or not a parent has the right to obtain such a waiver or release from the other parent (*i.e*., a disengagement of a gamete provider for the express purpose of avoidance of support obligations) on behalf of the child remains uncertain [89].

However, the 'right to procreate' has not yet gained the same legal traction that the right to avoid procreation has received [35,90], and procreational autonomy is only one model the Irish Courts could adopt in resolving a dispute over the fate of non-transferred embryos. This would require a difficult assessment of the rights of both parties involved (and potentiallly the rights of the embryo) in deciding who will control embryo disposition. Our judiciary is likely to balance the parties' rights in determining disposition of non-transferred embryos with consideration to precedents in the UK, U.S., and EHCR; such influences are likely to favour the party wishing to avoid procreation. Yet the need for statutory guidance is highlighted by the *Roche *case, as inconsistencies or omissions in treatment consent forms signed by Irish couples undergoing IVF are now left for medical providers to remedy at the clinic level. If this does not happen, then Irish Courts will expend considerable resources to resolve the resulting disputes. Indeed, 'the Irish Government has not provided a confidence-inspiring track record of legislatively dealing with complex and controversial problems raised either by reproduction or reproductive technologies. Policy and regulation is needed in the area of [assisted reproduction] and many observers of democracy assume that the public should have a positive role in debating desirable priorities for application' [91]. The vital public policy question therefore must be asked: Why has comprehensive regulation of IVF remained elusive in Ireland, despite our costly and impressive array of competent professional and academic agencies?

If it were established that there is a Constitutional right to procreate in Ireland, then prior decisions rendered elsewhere suggest that there is a superior correlative right *not *to procreate. In a dispute over the fate of non-transferred embryos, would the Constitutional protection of the right to life of the embryo (if it were defined as 'unborn' for the purposes of the Eighth Amendment/Article 40.3.3) then override the desire of a party seeking to avoid procreation, if that right 'inalienable and imprescriptable' was protected by Article 41 of the Constitution as 'superior to all positive law'? In the absence of any precedent here, the direction taken by Irish courts awaits the Supreme Court's judgment in the *Roche *case.

As the incidence of infertility rises, the number of IVF cycles will continue to grow. This trend, when added to the regrettable upsurge in marriage separation and divorce, cannot help but increase the potential for the involvement of Irish Courts in disputes involving non-transferred embryos. Although deciding the fate of non-transferred embryos in the wake of a relationship breakdown is an arduous task, the public policy consequences of ignoring the problem are undoubtedly more ominous. All branches of government in Ireland must act in a coordinated fashion and engage urgently on this far-reaching and profound issue with multi-generational ethical, political and legal ramifications.

## Competing interests

The authors declare that they have no competing interests.

## Authors' contributions

ESS and SEM developed this work collaboratively; both read and approved the final manuscript.
